# Outcomes of COVID-19 Admissions in the New York City Public Health System and Variations by Hospitals and Boroughs During the Initial Pandemic Response

**DOI:** 10.3389/fpubh.2021.570147

**Published:** 2021-05-11

**Authors:** Anant Dinesh, Taha Mallick, Tatiana M. Arreglado, Brian L. Altonen, Ryan Engdahl

**Affiliations:** ^1^NYC Health + Hospitals, Harlem Hospital, New York, NY, United States; ^2^Analytics & Reporting, Clinical Informatics, NYC Health + Hospitals, Harlem Hospital, New York, NY, United States; ^3^Population Health Analyst, NYC Health + Hospitals, New York, NY, United States; ^4^NYC Health + Hospitals Harlem and Woodhull Hospital, New York, NY, United States

**Keywords:** SARS-CoV-2, COVID-19, mortality, outcomes, variations, public hospitals, health systems, initial pandemic

## Abstract

**Introduction:** In the initial pandemic regional differences may have existed in COVID-19 hospitalizations and patient outcomes in New York City. Whether these patterns were present in public hospitals is unknown. The aim of this brief study was to investigate COVID-19 hospitalizations and outcomes in the public health system during the initial pandemic response.

**Methods:** A retrospective review was conducted on COVID-19 admissions in New York City public hospitals during the exponential phase of the pandemic. All data were collected from an integrated electronic medical records system (Epic Health Systems, Verona, WI). Overall, 5,422 patients with at least one admission each for COVID-19 were reviewed, with a study of demographic characteristics (including age, gender, race, BMI), pregnancy status, comorbidities, facility activity, and outcomes. Data related to hospitalization and mortality trends were also collected from City of New York website. These data often involved more than one facility and/or service line resulting in more location or treatment facility counts than patients due to utilization of services at more than one location and transfers between locations and facilities.

**Results:** Higher mortality was associated with increasing age with the highest death rate (51.9%) noted in the age group >75 years (OR 7.88, 95%CI 6.32–10.08). Comorbidities with higher mortality included diabetes (OR 1.5, 95% CI 1.33–1.70), hypertension (OR 1.62, 95% CI 1.44–1.83), cardiovascular conditions (OR 1.66, 95% CI 1.47–1.87), COPD (OR 1.86, 95% CI 1.39–2.50). It was deduced that 20% of all New York City COVID-19 positive admissions were in public health system during this timeframe. A high proportion of admissions (21.26%) and deaths (19.93%) were at Elmhurst Hospital in Queens. Bellevue and Metropolitan Hospitals had the lowest number of deaths, both in borough of Manhattan. Mortality in public hospitals in Brooklyn was 29.9%, Queens 28.1%, Manhattan 20.4%.

**Conclusion:** Significant variations existed in COVID-19 hospitalizations and outcomes in the public health system in New York City during the initial pandemic. Although outcomes are worse with older age and those with comorbidities, variations in hospitals and boroughs outside of Manhattan are targets to investigate and strategize efforts.

## Introduction

It has emerged that regional differences may have been present across COVID-19 hospitalizations and patient outcomes in New York City. Whether such patterns are also present in public health hospitals is largely unknown. Such information could be useful to public health infrastructure and to help understand the initial pandemic response to COVID-19 as well as to strategize future approaches should outbreaks occur. As prior studies have presented observations in private hospitals in the New York area (1), the aim of this brief study was to investigate COVID-19 hospitalizations and patient outcomes in the public health system during the initial pandemic response.

## Methods

A retrospective study was conducted on COVID-19 patients admitted to New York City public hospitals during the exponential phase of the pandemic. This public health system encompasses 11 hospitals in four different boroughs of New York City with two hospitals in Queens (Elmhurst Hospital and Queens Hospital), three in Manhattan (Bellevue Hospital, Metropolitan Hospital, and Harlem Hospital), three in Brooklyn (Kings County Hospital, Coney Island Hospital and Woodhull Hospital), and three in the Bronx (Jacobi Medical Center, Lincoln Hospital and North Central Bronx Hospital). The study was approved by Biomedical Research Alliance of New York (BRANY) and the Institutional Review Board. All data were collected from an integrated electronic medical records system (Epic Health Systems, Verona, WI). Demographics and outcomes including mortality on COVID-19 confirmed cases by real-time reverse transcriptase-polymerase chain reaction (RT-PCR) assay of nasopharyngeal swabs were analyzed. The data were collected for the period of 5 weeks from March 6th 2020 to April 9th 2020 comprising of the initial exponential phase, and completion of analysis was done by April 16th 2020. Variables were obtained including age, sex, race, body mass index, and pregnancy status, discharges, and mortality as well as discharge disposition. Deceased patients included patients who died during admission, also including mortalities in the emergency room at the time of presentation. We used descriptive statistics to characterize our study population. Continuous variables were calculated as mean, median, and ranges. Categorical variables were expressed as counts and percentages. We used Microsoft Excel 365 with add-in program Analysis ToolPak for statistical analysis. Univariate analysis was done using contingency tables and odds ratio with confidence interval was calculated to study the effect of different variables on mortality trends. Chi square independent tests (df = 2–11) were used to calculate *p*-value to determine the significance. A *P* < 0.05 was considered statistically significant, with significant Chi squared values dependent upon numbers of groups tested. Data related to hospitalization and mortality trends of New York City were collected from City of New York website COVID-19 data link^1^, which was compared with our data. City data on daily deaths and hospitalizations were used and compared with corresponding data generated for the public hospitals in this study. Data related to bed count including critical care beds for each hospital were obtained from New York State Department of Health website. These bed counts are the official approved beds for each hospital before the start of the pandemic, any increase in bed counts during the pandemic were not accounted for. Bed counts were used as a surrogate for resource and staff allocations. Due to overlapping services and change of service type between facilities, which impacted 21% of patients, amounts of overlap and re-admission had to be taken into account as part of the evaluation of boroughs and facilities evaluations, resulting in adjustments of services data based upon patients-visits activities data for H+H for the year 11/01/2019-10/31/2020 were evaluated for percent distribution of services per facility.

## Results

**Figure 1:**
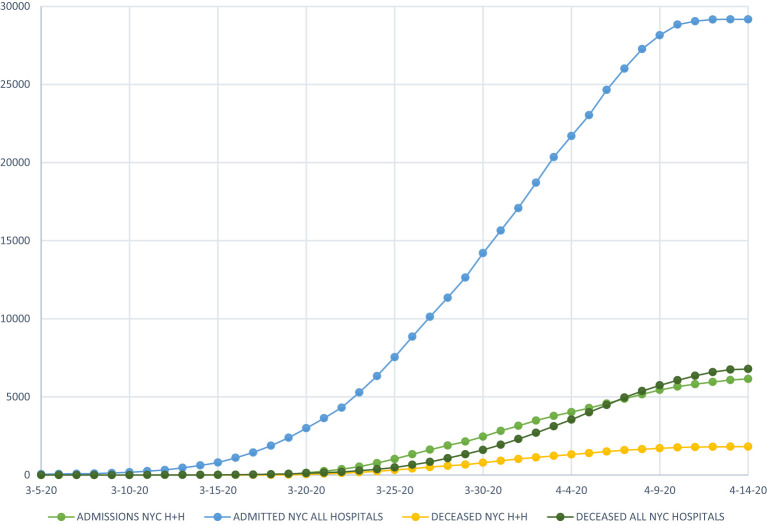
Comparison of admission and death trends of New York City public hospitals with all New York City hospitals.

**Figure 2:**
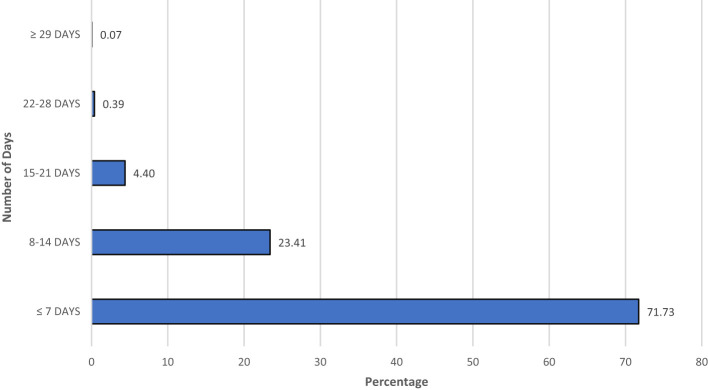
Length of stay in hospital in the initial pandemic of COVID-19 in positive patients in New York City public hospitals.

**Figure 3:**
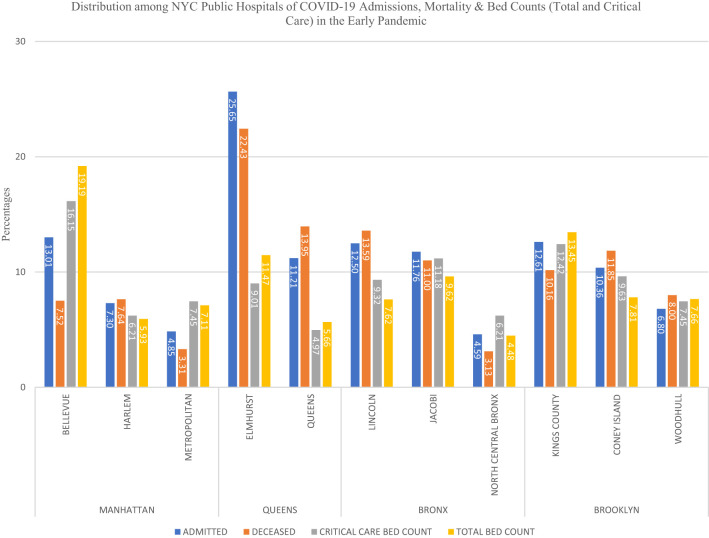
Distribution of COVID-19 hospitalized and deceased patients by specific hospital and borough in the initial pandemic in New York City.

A total of 18,147 patients were tested for COVID-19 during our study period and 11,599 (63.9%) were reported positive. Of these 11,599 patients, 5,422 patients (46.7%) were admitted to one of the eleven hospitals. Of the 5,422 patients admitted, 1,663 patients (30.6%) died and 2,158 (39.8%) were discharged, 1,601 patients were still admitted at the conclusion of the study. There were overall 1,521 (28.1%) inpatient deaths while 142 (2.5%) died in the emergency room at the time of presentation. The median age of the patients in our study population was 62 years (IQR 50–73 years). Ages 39–64 years contributed to 39% of the total admissions. Only 25 patients (0.46%) were <18 years of age. The majority of the admissions (78.7%) were noted as non-white including black or listed as other (which includes Hispanic and non-Hispanic populations). BMI was available for 3,235 of the admissions with the majority (67.2%) in the range of 25–39 kg/m^2^ (Table 1). Mortality was more prevalent in age group >75 years (Table 1). On univariate analysis of age groups, higher mortality was associated with increasing age and age group >75 years was associated with the highest mortality rate (OR 7.88, 95% CI 6.32–10.08). In the group <18 years of age just one death was seen (0.06% of all deaths). Hospitalization was more common in males (62.04%) than females (37.58%) with a slightly higher mortality rate noted in males (Tables 1, 2). Eighty-five patients (1.56%) were pregnant with no reported death at the time of analysis of this study. Comorbidities associated with higher mortality include diabetes (OR 1.5, 95% CI 1.33–1.70), hypertension (OR 1.62, 95% CI 1.44–1.83), and cardiovascular conditions (OR 1.66, 95%CI 1.47–1.87) and COPD (OR 1.86, 95% CI 1.39–2.50) (Table 2). From data available on City of New York website^1^, 20% of all COVID-19 positive admissions were in public health system during this timeframe (Figure 1). Median length of stay was calculated to be 5 days (IQR 2–9 days) among patients discharged or deceased and 72% of the study population had a hospital stay of <7 days (Figure 2). The distribution of the admissions during the COVID-19 pandemic and patient outcomes across the public hospitals were correlated with bed counts and ICU bed counts for each hospital are shown in Figure 3. Statistical differences for outcomes are shown in Table 2. Bed count information was from reference^2^.

**Table 1:** Demographics of patients admitted in the initial pandemic with COVID-19 positive results to New York City public hospitals.

	**Admissions**		**Deceased**	
**Characteristic**	***n* = 5,422**	**%**	***n* = 1,663**	**%**
<17 years old	25	0.46	1	0.06
18–44 years old	885	16.26	117	7.04
45–64 years old	2,146	39.43	450	27.06
65–74 years old	1,165	21.41	410	24.65
>75 years old	1,201	22.07	685	41.19
Female	2,045	37.58	604	36.32
Male	3,376	62.04	1,059	63.68
Unknown	1	0.02	0	0.00
**Race**				
White	495	9.10	201	12.09
Black	1,725	31.70	500	30.07
Asian	295	5.42	101	6.07
Others^*^	2,555	46.95	762	45.82
Unknown	382	7.02	107	6.43
Pregnant	85	1.56	0	0.00
BMI (kg/m^2^)^∧^	*n* = 3,235^∧^		*n* = 1,078^∧^	
≤18.4	79	2.44	29	2.69
18.5–24.9	697	21.55	244	22.63
25.0–29.9	1,135	35.09	372	34.51
30.0–39.9	1,039	32.12	324	30.06
>40.0	285	8.81	109	10.11

* *Includes Hispanic and non-Hispanic population*.

∧ *BMI only known for 4,313*.

**Table 2:** Deaths of admitted in the initial pandemic COVID-19 patients in New York City public hospitals.

**NYC Public Hospital**	**Deceased Percent (%)**	**Orig N**	**Expected, Eq Distrib**	**Readmissions**^**#**^	Adj pctgs^*^	**OR**	**95% CI**	***p*-value**
Bellevue	16.2	708	585	123	0.250	0.45	0.37–0.56	<0.001
Harlem	28.9	397	328	69	0.085	0.96	0.76–1.20	0.7
Metropolitan	17.7	264	218	46	0.115	0.51	0.37–0.70	<0.001
Elmhurst	23.9	1,396	1,153	243	0.050	0.74	0.64–0.85	<0.001
Queens	35.7	610	504	106	0.050	1.31	1.09–1.56	<0.001
Lincoln	31.1	680	562	118	0.100	1.06	0.89–1.26	0.06
Jacobi	26	640	528	112	0.115	0.83	0.69–1.0	0.22
North Central Bronx	20.1	250	206	44	0.030	0.59	0.43–0.81	<0.001
Kings County	23.8	686	566	120	0.150	0.73	0.61–0.89	0.01
Coney Island	33.7	564	466	98	0.005	1.20	0.99–1.44	0.00
Woodhull	35	370	306	64	0.050	1.27	1.01–1.58	0.00
Sums	100	6,565	5,421	1,144	1.000	–	–	–
**NYC Borough**								
Manhattan	20.4	1,317	1,125	192	0.445	reference	reference	reference
Queens	28.1	1,931	1,650	281	0.105	1.52	1.29–1.80	<0.001
Bronx	27.4	1,497	1,279	218	0.245	1.47	1.24–1.76	<0.001
Brooklyn	29.9	1,602	1,369	233	0.205	1.66	1.40–1.98	<0.001
Sums	100	6,347	5,422	925	1.000	–	–	–
**Comorbidity**								
Diabetes	33.6	1,828				1.50	1.33–1.70	<0.001
Hypertension	33.2	2,561				1.62	1.44–1.83	<0.001
End Stage Renal	29.3	140				1.06	0.74–1.54	0.74
cardiovascular disease	33.1	2,723				1.66	1.47–1.87	<0.001
Pulmonary disease	30.9	731				1.17	0.98–1.39	0.07
COPD	41.5	193				1.86	1.39–2.50	<0.001
Sums	100	8,176						

* *Adjusted percentages are for original population base rather than admission/readmission counts*.

# *Readmissions is the estimated number of patients entered more than once due to change in service location*.

The first part of Table 2 depicts data for patient-facility encounters, 1,144 of the 6,565 events (21%) were patients whose care was linked to more than one institution related to COVID-19 admission, such as a readmission, treatment provided by a second location, and/or transfer with readmission to a new facility. The most common reasons for these events could not be defined based upon the data provided by the EMR review. However, transfers included additional care to a new or special facility, i.e., patients transferred out of ICU to another location for a special care facility such as Bellevue for managing severely ill patients or facilities that provide a unique care for COVID-19 patients related to pregnancy or psychiatric care. The percent of admissions in the entire health system were noted to be highest in Elmhurst Hospital and Bellevue Hospital and similarly discharge rates were higher in these two hospitals, 52.8% (OR 1.84, 95% CI, 1.57–2.15, *p* < 0.001) and 51.9% (OR 1.77, 95% CI 1.57–1.99, *p* < 0.001), respectively. A high proportion of admissions (21.26%) and deaths (19.93%) were at Elmhurst Hospital. Differences in bed counts at two locations are noted, Bellevue in Manhattan and Elmhurst in Queens, with Bellevue having higher bed counts among the two. Examining variations in hospitals showed that Bellevue (16.2%, OR 0.45, 95% CI 0.37–0.56) and Metropolitan Hospitals (17.7%, OR 0.51, 95% CI 0.37–0.70) had the lowest number of deaths during this timeframe, both are in borough of Manhattan. Mortality in public hospitals in Brooklyn was 29.9% and Queens 28.1% and hospitals located in Manhattan 20.4%. The proportion of admissions was highest in Queens (30.4%) followed by Brooklyn (25.2%). Manhattan (20.8%) had the lowest proportion of admitted patients among public hospitals. While comparing data across different boroughs of New York City, Brooklyn had the lowest discharge rate (36.6%, OR 0.72, 95% CI 0.62–0.83, *p* < 0.001).

## Discussion

This study included 5,422 confirmed COVID-19 patients admitted in New York City public hospitals during the early phase of the pandemic. There are limited studies analyzing the public health system response during this timeframe. From data available from City of New York website^1^, we deduced that 20% of all COVID-19 positive admissions were in NYC public health system. Our study demonstrates the evidence of variation in patient outcomes among the 11 hospitals in NYC public health system, located in four different boroughs. In public hospitals in New York City, the overall mortality in admitted patients was demonstrated to be 28.1% during this time in the pandemic, which is slightly higher than other studies that have evaluated patient outcome in areas around the City (1) and in Manhattan alone (2) with mortality from 10–21%. Our data is in accordance with others (3, 4) with co-morbidities such as diabetes, hypertension, cardiovascular and pulmonary diseases contributing toward higher mortality. In a study conducted by the Northwell Health system (1) in New York involving 5,700 patients, mortality rate was seen to increase with age with much higher rates above 70 years and much lower rates in those below 30 years. Also noted in this study was a greater gender difference in mortality rate than that noted in our study with higher rates noted among males. Similar findings are noted in a study from Wuhan, China (3) which showed a statistically significant increase in mortality with age as well as a trend toward higher mortality in male patients. A study focusing on boroughs in this timeframe and hospitalization and death rates (5) due to COVID-19 found highest rates in the Bronx and lowest in Manhattan which may indicate an inverse correlation with affluence and socioeconomic status and a direct correlation with the proportion of ethnic minorities in different boroughs. Variability between different boroughs was also noted in our study with highest mortality rates noted in Brooklyn and Queens and lowest in Manhattan. Limitations of this study are the ones that are typical of the analysis of any initial pandemic. The COVID-19 status of other admitted patients in NYC public health system that were not included in the study was not known which might have led to underestimation of total number of cases. As we are still in an ongoing epidemic, we were not able to investigate a control population that may account for all the differences seen. In addition, as this study sole focus was on the initial pandemic response in the New York City health system, the outcomes of 1,601 patients who remained admitted during the study period were not incorporated into outcomes analysis. It is likely that disproportionate burden of COVID-19 has been borne by lower income and minority communities in New York City in the pandemic (5), such variations in the hospitals based on locations in different boroughs remain an important focus. Additional efforts and investigation of regional differences can likely help to strategize efforts that will help to improve outcomes of patients in public health system in the ongoing pandemic.

## Data Availability Statement

The raw data supporting the conclusions of this article will be made available by the authors, without undue reservation.

## Ethics Statement

The studies involving human participants were reviewed and approved by Biomedical Research Alliance of New York (BRANY). Written informed consent from the participants' legal guardian/next of kin was not required to participate in this study in accordance with the national legislation and the institutional requirements.

## Author Contributions

AD contributed to study design, data and analysis, and writing. TA and TM data collection, editing, and analysis. BA and RE study design, organization, analysis, and writing. All authors contributed to the article and approved the submitted version.

## Conflict of Interest

AD, TM, TA, BA, and RE were exployed by NYC Health + Hospitals.

## Footnotes

^1^https://www1.nyc.gov/site/doh/covid/covid-19-data.page

^2^https://profiles.health.ny.gov/hospital/view/103016#inspections
